# Food environment and obesity: a systematic review and meta-analysis

**DOI:** 10.1136/bmjnph-2023-000663

**Published:** 2024-04-22

**Authors:** Elisa Pineda, Jemima Stockton, Shaun Scholes, Camille Lassale, Jennifer S Mindell

**Affiliations:** 1 The George Institute for Global Health UK, Imperial College London, London, UK; 2 School of Public Health, Imperial College London, London, UK; 3 Research Department of Epidemiology and Public Health, University College London, London, UK; 4 Barcelona Institute for Global Health (ISGlobal), Barcelona, Spain; 5 CIBER Physiopathology of Obesity and Nutrition (CIBEROBN), Carlos III Health Institute (ISCIII), Madrid, Spain

**Keywords:** Malnutrition

## Abstract

**Background:**

Obesity is influenced by a complex, multifaceted system of determinants, including the food environment. Governments need evidence to act on improving the food environment. The aim of this study was to review the evidence from spatial environmental analyses and to conduct the first series of meta-analyses to assess the impact of the retail food environment on obesity.

**Methods:**

We performed a systematic review and random-effects meta-analyses, focusing on geographical–statistical methods to assess the associations between food outlet availability and obesity. We searched OvidSP-Medline, Scielo, Scopus and Google Scholar databases up to January 2022. The search terms included spatial analysis, obesity and the retail food environment. Effect sizes were pooled by random-effects meta-analyses separately according to food outlet type and geographical and statistical measures.

**Findings:**

Of the 4118 retrieved papers, we included 103 studies. Density (n=52, 50%) and linear and logistic regressions (n=68, 66%) were the main measures used to assess the association of the food environment with obesity. Multilevel or autocorrelation analyses were used in 35 (34%) studies. Fast-food outlet proximity was positively and significantly associated with obesity (OR: 1.15, 95% CI: 1.02 to 1.30, p=0.02). Fresh fruit and vegetable outlet density and supermarket proximity were inversely associated with obesity (OR: 0.93, 95% CI: 0.90 to 0.96, p<0.001; OR: 0.90, 95% CI: 0.82 to 0.98, p=0.02). No significant associations were found for restaurants, convenience stores or any of the body mass index measures.

**Conclusions:**

Food outlets which sell mostly unhealthy and ultra-processed foods were associated with higher levels of obesity, while fruit and vegetable availability and supermarket accessibility, which enable healthier food access, were related to lower levels of obesity. The regulation of food outlets through zoning laws may not be enough to tackle the burden of obesity. Regulations that focus on increasing the availability of healthy food within stores and ensure overall healthy food environments require further attention.

**PROSPERO registration number:**

CRD42018111652.

**WHAT IS ALREADY KNOWN ON THIS TOPIC**

- The food environment is a recognised key determinant for the prevention of obesity and other diet-related non-communicable diseases (NCDs). Multiple studies have identified inconsistent findings regarding the association between elements of the retail food environment and obesity. Variability in geographical and analytical methods has been pointed out as a potential cause for these discrepancies.

**WHAT THIS STUDY ADDS**

- This systematic literature review and meta-analyses consolidates all the evidence and effect sizes to determine which elements of the retail food environment have the greatest impact on obesity. It stratigically considers elements of the retail food environment, along with geographical and statistical methods to provide increased statistical power, accuracy, and a comprehensive summary of findings regarding the association of the food environment with obesity.

**HOW THIS STUDY MIGHT AFFECT RESEARCH, PRACTICE OR POLICY**

- The evidence generated from this systematic review and meta-analyses can serve as a foundational tool for policymakers and researchers in developing programmes and interventions for the prevention of obesity and other diet-related NCDs. This study offers a quantitative and visual guide for identifying the retail food environment elements that require greater focus in strategies aimed at tackling obesity.

## Introduction

### The retail food environment and obesity

Obesity, a critical risk factor for non-communicable diseases (NCDs), is prevalent in countries across all income levels, including low-, middle- and high-income nations.^1 2^ Its prevalence is shaped by a complex array of determinants, notably the retail food environment and advertising landscapes.^3^ Modern food environments are marked by the widespread availability and promotion of energy-dense, nutrient-poor foods.^4^ For instance, the increase in food retailers has contributed to a significant rise in calorie availability, facilitating greater access to a wide array of food choices.^5^ To combat structural overconsumption and curb the obesity epidemic, policy interventions must be enacted, even in the face of commercial interests. However, the specific influence of food environments on obesity, as distinct from individual behaviour, remains poorly defined.^6 7^ There is a scarcity of evidence identifying the exact elements of food environments that contribute to obesity and could be targeted for change.^3 4 8^ This review aims to enhance understanding of the analytical methods required to dissect the various components of the modern retail food environment in relation to obesity and to assess the impact of retail food environments on obesity levels.

### Analysing the retail food environment

Spatial analysis, leveraging Geographic Information Systems (GIS), has become instrumental in exploring the interplay between the environment and health outcomes. It particularly aids in investigating the food environment by mapping the locations of food stores, examining their spatial distribution and assessing their impact on obesity and population health. This approach enables the study of how the proximity and density of food outlets relative to residential areas influence access to healthy versus unhealthy food options, thereby identifying key environmental factors and protective measures against obesity through spatial patterns.^9–12^

### Previous literature reviews

Previous literature reviews on the relationship between the retail food environment and obesity have underscored methodological issues that may affect the analysis and interpretation of how food environments influence health and dietary outcomes. There is a recognised need for precise, comprehensive evaluations, including standardised and validated measurement techniques and diverse approaches to assessing the retail food environment, as current methods exhibit considerable variability.^12–14^ Essential aspects of retail food environment research involve confirming the location and type of food outlets through store audits (ground truthing),^13^ considering the confounding effects of socioeconomic status^14 15^ and using longitudinal studies to observe changes in the retail food environment and dietary choices over time.^15 16^

Despite numerous studies investigating the retail food environment’s impact on obesity, systematic reviews and meta-analyses are scarce.^17–20^ Previous analyses have often been restricted to specific regions or populations, with limited attention to the methodologies for measuring the retail food environment.^17–20^ This paper undertakes a systematic review and meta-analyses to synthesise available evidence on the retail food environment’s role in obesity and diet-related NCDs, aiming to pinpoint elements that could be targeted by policy interventions. Furthermore, it critically assesses the methodological strategies used to study the global impact of the retail food environment on obesity.

### Obesity and the food environment

The food environment encompasses physical, economic, political and sociocultural factors affecting dietary choices.^21^ Glanz *et al.*’s^22^ model suggests that dietary intake is shaped by policy, environment, individual and behavioural factors. This includes the community nutrition environment (types of food stores, locations, and availability), which in this study we refer to as the 'retail food environment'; organisational settings (neighbourhood, school, workplace); and consumer aspects (food availability, placement, pricing, promotions, nutrition labelling). Key attributes defining the food environment are geographical access, availability, affordability and advertising.^23–25^ While various factors contribute to obesity, environmental and policy measures can significantly improve the food environment, leading to widespread dietary changes and reduced obesity and disease rates.^26^

## Methods

We performed a systematic review and meta-analyses to assess the association of the retail food environment with adult obesity and to evaluate the geographical and statistical methods used. PRISMA (Preferred Reporting Items for Systematic Reviews and Meta-Analyses) guidelines were followed (online supplemental figure S1). Search results were screened by two reviewers for eligibility. The review was registered in PROSPERO as CRD42018111652.

10.1136/bmjnph-2023-000663.supp1 Supplementary data

### Literature search strategy

We conducted a literature search on 31 January 2022, spanning papers published from 1946 onwards, to identify studies focusing on the impact of the retail food environment on obesity through spatial analysis. Using OvidSP-Medline, Scopus and Google Scholar databases, we structured the search around three primary themes: the retail food environment, obesity and spatial analysis. Initially, each theme was explored individually, and subsequently, we employed the ‘AND’ operator to search them concurrently. Using the Population, Intervention, Control, Outcome (PICO) framework (online supplemental table S1) for eligibility assessment,^27^ we considered publications examining the influence of the retail food environment on adult obesity or body mass index (BMI) for inclusion in our systematic literature review and meta-analyses.

Our literature search strategy involved MeSH words, Boolean search terms and proximity searching characters ($, *, W, #) on Medline (OvidSP, 1946–current: 31 January 2022). The terms covered diverse aspects such as buffer, chain, convenience, density variations (denoted by densit*), desert, distance, eating habits (indicated by eat$), environmental factors, farmers’ markets, fast food, geography, geolocation, geospatial analysis, GIS (geographic information systems), global, grocery stores, increase, index, location, markets, access, provision, proximity, restaurants, retail, spatial considerations, stores, supermarkets, supply, BMI (body mass index), body mass, nutrition, obesity, overweight, positional factors, weight gain and overeating. Additionally, the search extended to Scopus and Google Scholar using the query “(ALL (obesity) AND ALL (food environment OR convenience store OR food retail) AND ALL (GIS OR spatial analysis OR geographic information systems))” as of 31 January 2022.

### Risk of bias and quality assessment criteria

Risk of bias and quality were evaluated using a weighted quality score derived from the Cochrane risk-of-bias tool, the systematic review data collection procedures from The Guide to Community Preventive Services^28^ and the food environment quality assessment by Williams *et al*.^29^ Nine criteria were assessed: population representativeness, outcome validity, exposure representativeness, exposure source, retail food environment assessment method, physical activity assessment, study design, statistical methods and data temporality. Studies received one point for each criterion met (online supplemental table S2).

### Spatial and statistical methods and study design appraisal

Study design, statistical methods and models were explored and assessed according to their consideration of spatial clustering,^30^ and according to their inclusion of confounders.

### Meta-analysis

We performed random-effect meta-analyses to explore the link between the retail food environment and obesity, analysing data from various outlets including fast-food restaurants, convenience stores, supermarkets and farmers’ markets. We evaluated the retail food environment using density, proximity and the Retail Food Environment Index (RFEI)—the ratio of unhealthy to healthy food outlets. Our analyses focused on ORs for categorical outcomes and beta-coefficients (β) for continuous variables, combining similar measures for meta-analyses. We assessed the impact of the retail food environment on adult BMI (β) and obesity prevalence (ORs), selecting the most relevant estimate from studies providing multiple results to ensure observations remained independent. Only models adjusted for confounders were included. For comparability, we considered data within 1 mile buffers or equivalent, representing walkable distances. In longitudinal studies, the most recent data were used. When results were stratified by sex and socioeconomic position (SEP), we chose observations based on the largest sample size or prioritised women and low-income groups if sizes were equal. We reported effect sizes and 95% CIs for each study, using Stata V.16.0 for all statistical analyses.^31^

## Results

We retrieved 4118 studies, and after applying inclusion and exclusion criteria, retained 103 articles yielding 526 data points (online supplemental figure S1). These were categorised by statistical measure, geographical measure and food outlet type, with 437 data points used in meta-analyses and meta-regression. The analysis covered 16 countries, with 90% of the studies from high-income countries: 1 from Africa, 5 each from Asia, Latin America and Australia, 14 from Europe and 74 from North America, spanning from 2004 to 2021, predominantly between 2011 and 2017 (n=54, 52%) (online supplemental table S3).

In terms of retail food environment measures, 52 (50%) studies evaluated density, 21 (20%) proximity, 3 (3%) both, 4 (4%) the RFEI or variants and 15 (15%) other measures like ratio and diversity. Most studies (n=77, 75%) assessed one geographical measure, 20 (19%) evaluated two and six (6%) assessed up to three. From the 526 data points that were extracted from all studies, fast-food outlets were the most examined (n=166, 32%), followed by supermarkets (n=102, 19%), restaurants (n=101, 19%) and convenience stores (n=61, 12%), fresh fruit and vegetable stores (n=17, 3%), grocery stores (n=14, 3%), specialty stores (n=8, 2%), supercentres (n=5, 1%), and farmers’ markets (n=4, 1%). A majority of the studies, 61% (n=63), accounted for walkability or physical activity as a confounder (online supplemental table S4).

Associations varied by geographical area, underscoring the need for representative geographical selection. For example, Fan *et al* 32 found different associations between restaurants and obesity for men at the census tract level and for women at the block level. However, 64% (n=66) of studies did not perform ground truthing or verify retail food environment data (online supplemental table S4).

### Statistical and geographical methods

Of the studies analysed, 68 (66%) applied linear or logistic regression, while 35 (34%) used multilevel modelling or methods accounting for spatial factors and clustering (online supplemental table S3). In terms of data sources for food outlet locations, 39 (38%) used government databases, 27 (26%) commercial databases, 14 (14%) conducted ground truthing, 23 (22%) employed various methods and 1 (1%) did not disclose their source. Among the studies employing multilevel modelling or spatial considerations, 26 (74%) identified positive correlations between the presence of food retailers selling foods high in fat, sugar and salt (HFSS) and obesity rates (online supplemental table S3).

### Study design

Of the 89 cross-sectional studies analysed, 59 (66%) discovered a correlation between obesity and food retailers specialising in unhealthy foods and beverages, such as convenience stores and fast-food outlets. Among the 14 longitudinal studies, half revealed a significant link between the presence of unhealthy food outlets and obesity (refer to online supplemental tables S3 and S4 for detailed findings).

### Quality and bias assessment of studies

The mean quality score of the studies was low, at 4 out of 9 points, with the highest being 7.^33 34^ Key limitations included the reliance on cross-sectional designs, the failure to account for clustering or to apply spatial methods in 30 (29%) studies, reliance on self-reported height and weight data in 34 (33%) studies and the use of inappropriate statistical methods in 43 (42%) studies (online supplemental table S5). Studies deemed to have a high risk of bias were excluded from the meta-analyses.

### Meta-analysis

In the meta-analyses conducted, significant heterogeneity was observed across the studies, stemming from variations in statistical methods, study designs, stratification by gender and ethnicity, geographical measures of the retail food environment, classifications of food outlets and the definitions used to measure or define obesity, thereby limiting the robustness of the pooled analyses. Despite these variances, the majority of the studies used BMI, derived from measured height and weight, as a primary indicator, reporting it either as a continuous variable (kg/m^2^) or in categorical terms (overweight or obesity). However, there was a notable scarcity of studies disaggregating outcome data by critical demographic factors such as age group, gender, ethnicity or SEP, which is pivotal considering the diverse exposure to retail food environments experienced by these groups.^35^ Results of the meta-analyses are presented below by measure of the retail food environment (ie, density and proximity) and statistical measures (ORs and Beta-coefficients─in the supplemental material).

**Figure 1:**
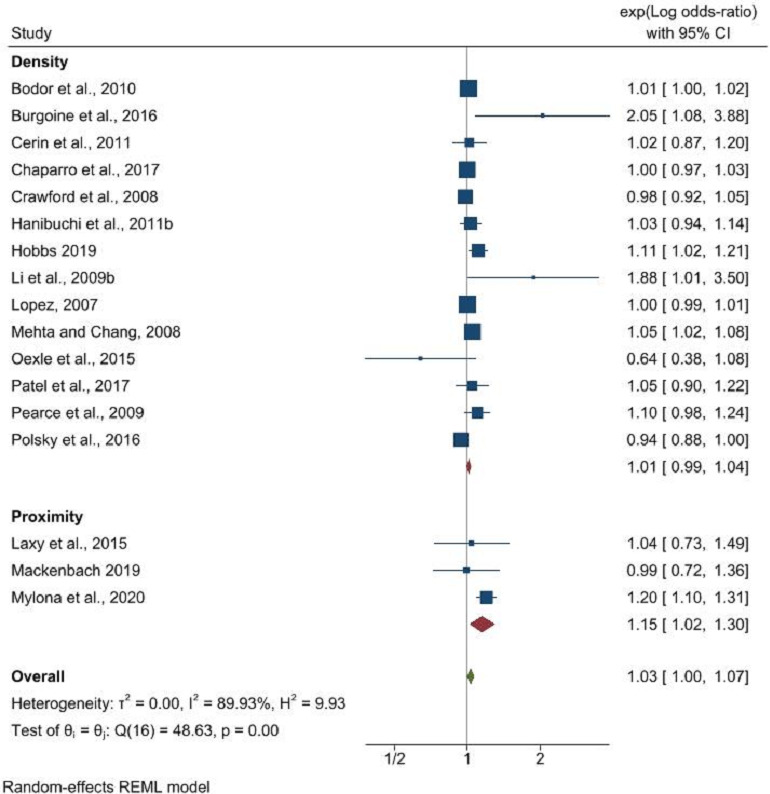
Fast-food outlet density and proximity and its association with obesity. REML, Restricted Maximum Likelihood.

**Figure 2:**
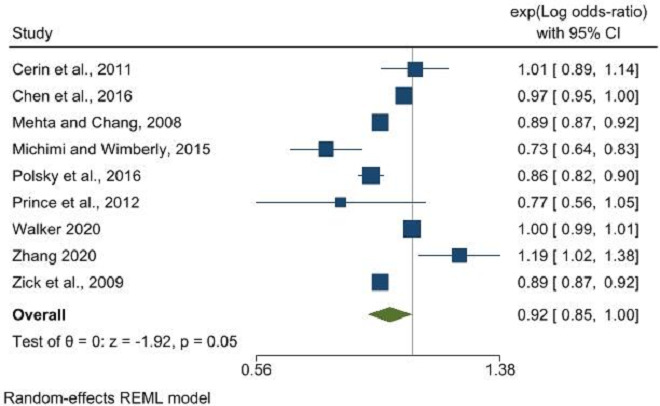
Restaurant density and its association with obesity. REML, Restricted Maximum Likelihood.

**Figure 3:**
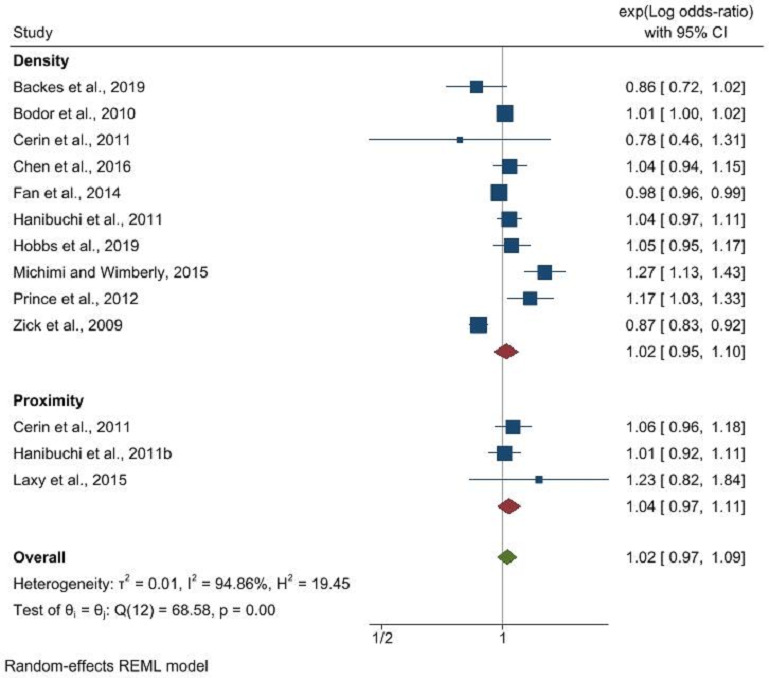
Convenience store density and proximity and its association with obesity. REML, Restricted Maximum Likelihood.

The findings revealed that the density of fast-food outlets did not significantly influence obesity rates (OR: 1.01, 95% CI: 0.99 to 1.04, p=0.18), in contrast to proximity to fast-food outlets, which showed a significant association with obesity (OR: 1.15, 95% CI: 1.02 to 1.30, p=0.02) (figure 1). Restaurant density’s correlation with obesity was marginally significant (OR: 0.92, 95% CI: 0.85 to 1.00, p=0.05), yet the literature lacked sufficient data to evaluate the impact of restaurant proximity (figure 2). No significant relationship was identified between the density of convenience stores and obesity (OR: 1.02, 95% CI: 0.95 to 1.10, p=0.64), and a similar non-significant trend was observed for proximity to convenience stores (OR: 1.04, 95% CI: 0.97 to 1.11, p=0.31) (figure 3).

**Figure 4:**
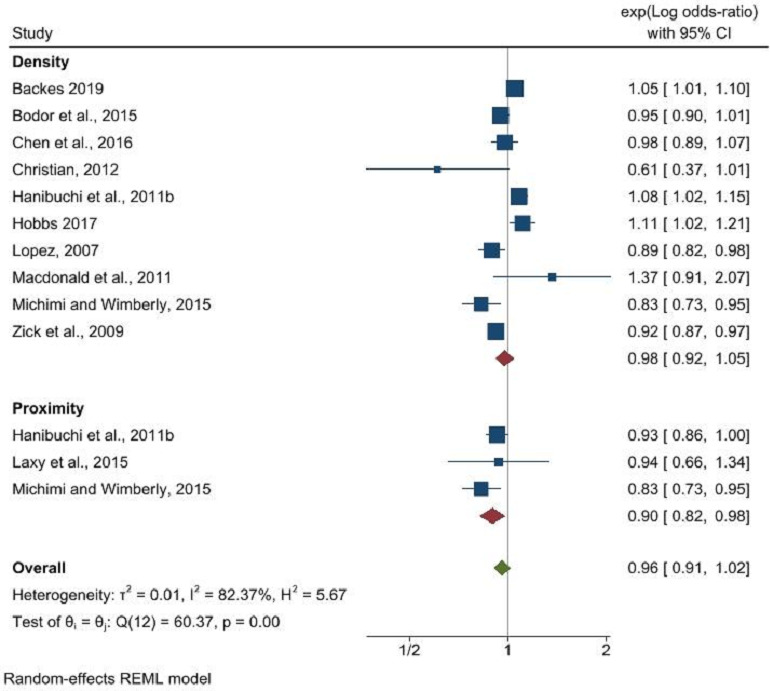
Supermarket density and proximity and its association with obesity. REML, Restricted Maximum Likelihood.

**Figure 5:**
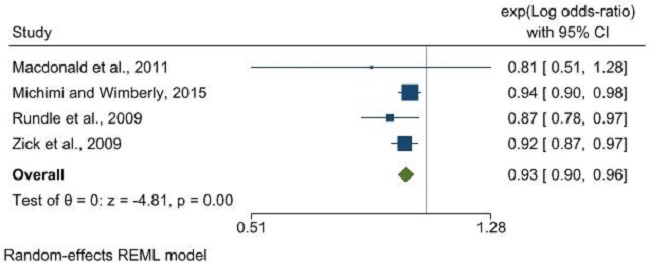
Fruit and vegetable store density and its association with obesity. REML, Restricted Maximum Likelihood.

**Figure 6:**
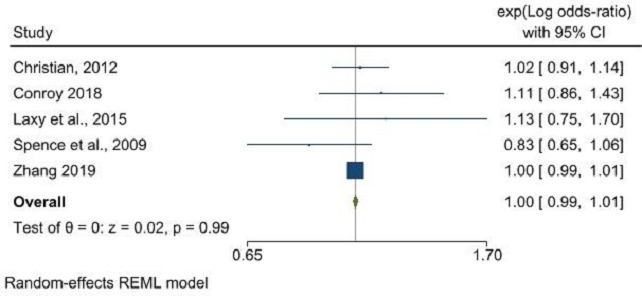
Retail Food Environment Index (RFEI) and its association with obesity. REML, Restricted Maximum Likelihood.

Furthermore, supermarket density did not show a significant relationship with obesity (OR: 0.98, 95% CI: 0.92 to 1.05, p=0.53), whereas a significant inverse relationship was evident between supermarket proximity and obesity (OR: 0.90, 95% CI: 0.82 to 0.98, p=0.02) (figure 4). An inverse association was also noted between the density of fresh fruit and vegetable stores and obesity (OR: 0.93, 95% CI: 0.90 to 0.96, p<0.001) (figure 5), though data were insufficient to assess the impact of proximity to these outlets. The RFEI did not reveal any significant associations with obesity (OR: 1.00, 95% CI: 0.99 to 1.01, p=0.99) (figure 6), and BMI as a continuous variable showed no association with any type of food outlet, indicating a nuanced and complex relationship between the retail food environment and obesity (online supplemental figures S2–S7).

## Discussion

The results of our systematic review and meta-analyses indicate a nuanced relationship between the retail food environment and obesity. Results for the association between the retail food environment and obesity varied significantly by type of food outlet, statistical measure and geographical measure. However, the pooled effect sizes show that proximity of fast-food outlets was associated with a higher risk of obesity, while proximity of supermarkets and fresh fruit and vegetable stores was associated with a lower risk of obesity.

Previous research highlights the crucial role of fruit and vegetable availability and affordability in fostering healthy eating habits and preventing obesity and chronic diseases.^36^ Conversely, fast-food outlets predominantly offer ultra-processed foods—industrially processed items rich in fat, salt and/or sugar—whose consumption is associated with increased risks of obesity and chronic conditions.^37^

The observed phenomenon can be attributed to the ease of access to different types of food outlets and their impact on dietary choices. Fast-food outlets, often closer to residential areas or on the pathways from school or the office to home, provide convenient access to high-calorie, processed foods, which can contribute to higher obesity rates among nearby residents.^14^ Conversely, supermarkets, which are sometimes located further from residential areas, offer a broader range of healthier food options. When supermarkets are closer, it encourages the purchase and consumption of healthier foods, potentially reducing obesity risk.^38^ This highlights the significant role of the retail food environment accessibility in influencing dietary behaviours and obesity prevalence.

In addition, socioeconomic area level may play a critical role in this context by influencing both access to and choices within the retail food environment.^39^ Individuals living in lower socioeconomic areas may have more limited access to supermarkets offering a variety of healthy options due to cost or proximity, leading to a reliance on closer, often less expensive fast-food outlets.^39^ This disparity can result in dietary patterns that contribute to higher obesity rates in these populations, underscoring the need for targeted interventions to improve access to healthy food options across all socioeconomic groups.

Importantly, while geographical measures such as proximity and density provide insights into the retail food environment or built food environment, they do not capture the complexities *within* food outlets that influence consumer choices. The 'in-store food environment', encompassing product placement, promotion strategies and food layout, plays a pivotal role in shaping dietary habits. Studies have demonstrated that strategic placement of healthy food options at eye level or in prominent store locations can significantly influence consumer purchases towards healthier choices.^40–43^

A comprehensive approach, addressing both the proximity of various food outlet types and the intricate details of the in-store food environment, is essential for devising effective public health interventions aimed at reducing obesity. Future research and policy efforts should consider these dimensions of the food environment to develop more nuanced and impactful strategies for obesity prevention.

The UK is a pioneer in regulating the food environment, having introduced legislation to restrict the promotion and placement of HFSS foods within retail settings, both online and physical.^44^ This legislation targets the influence of food retailers on consumer choices, particularly aiming to reduce the impact of price promotions on children’s food preferences by limiting promotions and strategic placement of HFSS products. This is a crucial step in promoting healthier eating habits and combating obesity and related health issues.

Additionally, in high-income countries, zoning powers allow local authorities to regulate food outlets’ location, and healthy food carts have been effectively deployed in urban areas to increase access to nutritious food.^18^

Studies on the food environment can inform the creation of improved land use and public health policies, mitigating the negative effects of local food and nutrition environments on population health^45^ Effective obesity reduction efforts should include policies or regulations to limit the availability of low-quality food in neighbourhoods, schools and other sensitive areas. However, the relationship between food outlets and obesity has shown inconsistent results, underscoring the need for solid evidence to guide government actions on enhancing the food environment.

This research significantly advances the evidence^18–20^ by integrating a systematic review with meta-analyses to explore the retail food environment’s influence on obesity and BMI. This dual approach, not previously used for this topic, integrates geographical and statistical analyses and offers a comprehensive analysis of the relationship between food outlet types, BMI and obesity. Furthermore, this study is distinct as it includes analyses that employ spatial methodologies to explore the retail food environment’s components and their correlation with obesity, providing a comprehensive evidence base for policy formulation aimed at enhancing public health.

### Implications for policymakers and urban planners

The observed association between fast-food outlet proximity and increased obesity risk emphasises the need for zoning regulations to manage their density in residential areas, schools and communal spaces. This strategic intervention becomes crucial in mitigating the obesity crisis. Our study discerns variations in associations among different food outlet types. While proximity of fast-food outlets correlates positively with obesity, proximity of supermarkets and fresh produce stores demonstrates an inverse relationship. Urban planners can influence health outcomes by strategically placing health-promoting outlets in residential areas, aligning with the concept of fostering a ‘healthy food environment’.

Beyond reaffirming existing knowledge, our study introduces novel insights into nuanced relationships between specific food outlets and obesity risk. Policymakers and urban planners can leverage this information to refine existing zoning laws based on prevalent food outlet types.

Our analysis also reveals a gap in the assessment of in-store food environments. Policymakers should focus on internal dynamics, implementing regulations targeting the arrangement and promotion of food items within stores to encourage healthier choices. Moreover, they should engage with town planners, health professionals and community representatives to develop comprehensive strategies. Collaborative efforts can lead to urban spaces that limit the impact of detrimental food outlets and food choices while promoting health and well-being. This aligns with the broader goal of fostering healthier communities, emphasising the importance of continued research and dialogue between academia and policymakers.

### Strengths and limitations

This study’s primary strength lies in its comprehensive systematic search strategy, which involved querying multiple databases, imposing no publication date restrictions and conducting searches in two languages. Additionally, it uniquely explored and assessed geographical measures and statistical methods within a systematic literature review context and conducted a risk-of-bias assessment to objectively evaluate the reviewed literature.

By incorporating spatial analysis, this study addressed gaps in previous literature by elucidating the impact of food outlets’ geographical distribution on obesity rates. This approach enabled the identification of spatial patterns and correlations potentially overlooked in traditional epidemiological studies, thereby providing insight into the obesogenic environment.

Spatial analysis also enhanced the meta-analyses by facilitating the integration and comparison of findings from studies across different geographical scales and settings, thereby bolstering the robustness of our conclusions. This rigour in methodology supported evidence synthesis, offering a detailed overview of the retail food environment’s role in obesity.

Through a detailed spatial analysis, our study not only corroborates the significance of geographical factors in obesity prevalence but also underscores the need for targeted public health interventions. By pinpointing areas with high concentrations of unhealthy food outlets relative to healthy ones, policymakers and urban planners can devise more effective strategies aimed at improving the food environment and, subsequently, public health.

However, the study has limitations. The review focused on obesity in the adult population because of the diverse reviews already focused on children, and because of the important role that adults play in food outlet selection within a family setting. Focusing on adult populations is critical for chronic disease prevention and successful ageing. Only studies based on neighbourhood, rural or urban environments were considered. Studies that did not include an objective measure of obesity such as BMI via measured height and weight were excluded. However, many studies that used BMI and other measures of diet and obesity were considered. The identified exposures, measures and outcomes included in this study were the most reported in the literature. Although this may exclude other important obesity-related outcomes (eg, adiposity, fat mass, diet), focusing on BMI and obesity allowed a wider comparison between studies and could facilitate translation into policies and actions to regulate and improve the food environment.

## Conclusion

Despite significant methodological diversity among the studies reviewed, the literature consistently identifies the food environment as a crucial factor in preventing obesity. Regions characterised by abundant fast-food outlets, limited supermarket access and scarce fresh fruit and vegetable stores tend to have higher obesity rates. While regulating access to healthier food options is necessary, it may not suffice to combat obesity on its own. Comprehensive strategies are also needed, including regulation of the in-store availability of unhealthy foods and the promotion of a food environment that supports healthy and affordable diets.

## Data Availability

All data relevant to the study are included in the article or uploaded as supplementary information.
