# Supplementary material for: Food environment and obesity: a systematic review and meta-analysis
Source: BMJ Nutr Prev Health. 2024 Apr 22;7(1):204–11. doi: 10.1136/bmjnph-2023-000663 (PMC11221287; doi:10.1136/bmjnph-2023-000663)

BMJ Publishing Group Limited (BMJ) disclaims all liability and responsibility arising from any reliance placed on this supplemental material which has been supplied by the author(s)

The Food Environment and Obesity: A Systematic Review and Meta-analysis

Supplementary Material

Contents

Supplementary Material 1
1. PRISMA Flow Diagram 2
   Included 2
   Eligibility 2
   Screening 2
   Identification 2
2. Eligibility criteria 3
Table S1. Eligibility criteria for publications relevant to the review using PICO 3
3. Risk of bias 4
4. General characteristics of reviewed studies 5
5. Risk of bias assessment 19
6. Food outlet density and proximity and its association with BMI 24

1. PRISMA Flow Diagram

Figure S1. PRISMA Flow Diagram

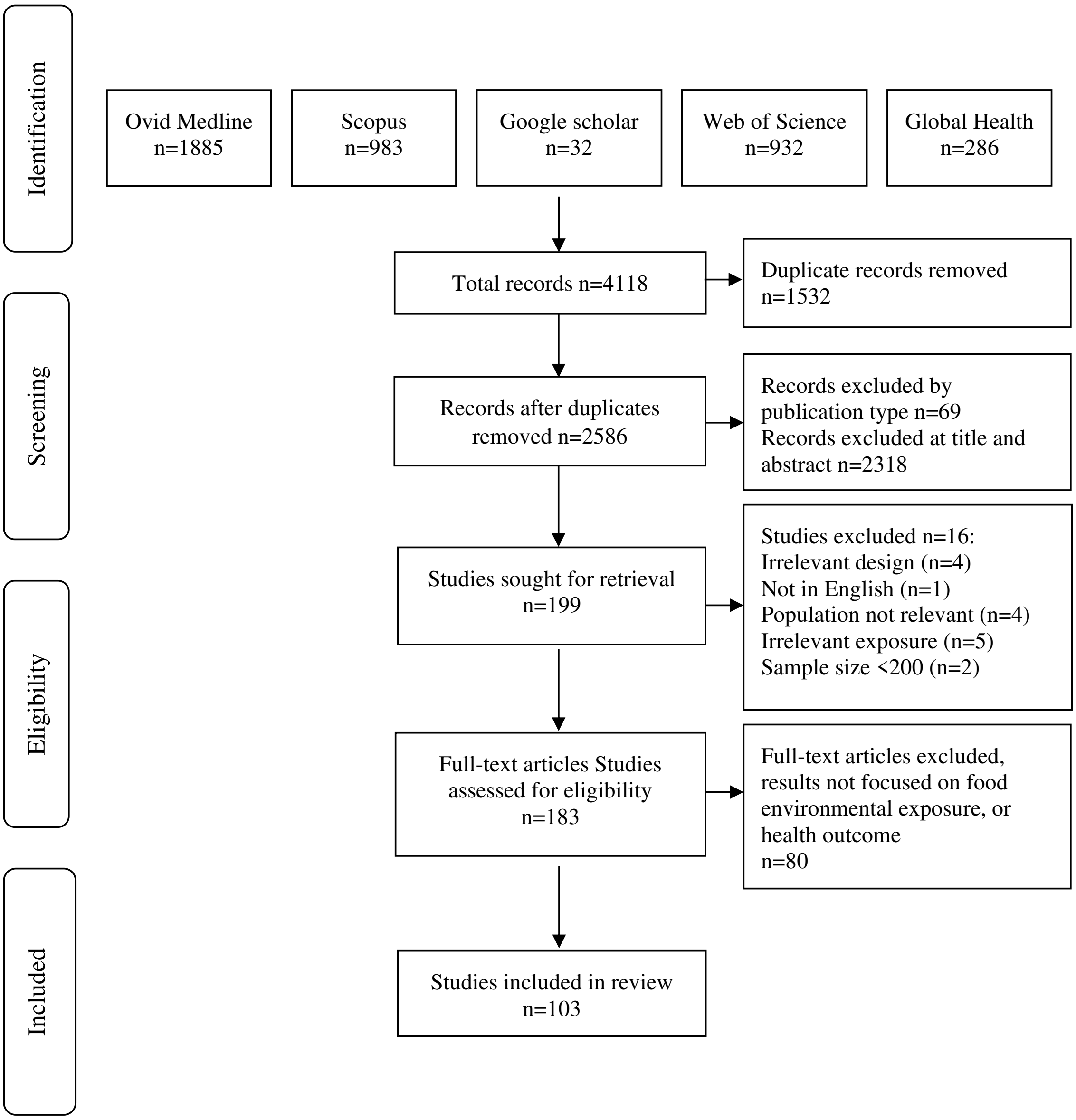

2. Eligibility criteria

Table S1. Eligibility criteria for publications relevant to the review using PICO

**Population**	General adult population, excluding populations with comorbidities.
**Intervention/ exposure**	Assessment of the retail food environments at any geographical level (e.g., census tract area, postal code, county, city, etc)
**Control**	Not applicable/areas with no food environment
**Primary Outcomes**	Obesity related outcomes (e.g., BMI, obesity prevalence, adiposity, etc.)

3. Risk of bias

Table S2. Risk of bias and quality criteria assessment for reviewed studies

Criterion	Meeting criteria (Score 1)	Not meeting criteria (Score 0)
Population	Population was randomly selected, and proper sampling methods were undertaken to select a representative population according to the study's aims.	Population was selected using telephone surveys.
Outcome (BMI)	Measured weight and height or validated data.	Self-reported or non-validated data used.
Exposure (food environment)	> 2 types of food establishments were studied.	≤ 2 types of food establishments were studied
Food outlet data source	Validated or trustworthy source (e.g., up-to-date government database) used or ground truthing was undertaken).	Data were not validated.
Spatial analysis method	≥ 2 methods were employed.	Only one method.
Physical activity (PA)	PA or walkability was considered in the model.	Neither PA nor walkability considered.
Study design	Longitudinal studies.	Cross-sectional or ecological design
Statistical method	Use of Moran's I, multilevel analysis, geographic weighted regression analysis or any other method that considered space or clustering as an important variable of influence.	Linear regression methods which do not consider space or clustering.
Data temporality	Health and food outlet data were from same year.	Health and food outlet data varied in collection time.

4. General characteristics of reviewed studies

Table S3. General characteristics and descriptive summary of reviewed studies

Reference	Country	Sample population	Physical activity consideration	Statistical Method	Significant findings
**Cross-sectional studies**					
**Abbott et al., 2014**	Australia	1,819	Yes	Linear regression model	Yes
**Adachi-Mejia 2017**	USA	2,025	Yes	Multiple regression models	Mixed
**Ahern et al., 2011**	USA	USA population	Yes	Linear regression	Yes
**Albalawi et al., 2020**	UK	456,079	No	Multiple linear regressions	No
**Backes et al., 2019**	Brazil	1,096	No	Multilevel Poisson regression models (with robust standard error)	No
**Bodor et al., 2010**	USA	3,925	Yes	Hierarchical linear models	Yes
**Burgoine 2017**	UK	9,702	Yes	Multiple linear regression	Yes
**Burgoine 2018**	UK	51,361	Yes	Multivariable linear and binomial logistic regression	Yes
**Cerin et al., 2011**	USA	274	Yes	GLM with binomial variance and logit link functions	Yes
**Chaparro et al., 2017**	USA	1,041	No	Multilevel logistic regression	No
**Chen et al., 2010**	USA	844,187	No	OLS spatial diagnostic test on regression residual of non-spatial model	Yes
**Chen et al., 2013**	USA	3,550	Yes	OLS	Yes
**Chen et al., 2016**	USA	25,023	No	Multilevel	Yes
**Chen et al., 2019**	USA	20,897	Yes	Ordinary least squares linear regression (evaluated by Moran’s I index) global ordinary least squares regression local geographically weighted regression	Yes
**Chen et al., 2020**	USA	20,897	No	Path analysis	Yes

Reference	Country	Sample population	Physical activity consideration	Statistical Method	Significant findings
**Christian et al., 2012**	USA	121	Yes	Multivariate logistic regression	No
**Conroy et al., 2018**	USA	102,906	Yes	multivariable linear regression multivariable multinomial logistic regression	No
**Cooksey-Stowers et al., 2017**	USA	3,108	Yes	OLS	Yes
**Crawford et al., 2008**	Canada	684	Yes	Bivariate logistic regression	No
**Dake et al., 2016**	Ghana	657	Yes	Multilevel	Yes
**Drewnowski et al., 2012**	USA	1,304	No	Modified Poisson regression	No
**Drewnowski et al., 2014a**	USA-France	9,291	No	Modified Poisson regression w/ robust error variance	No
**Dunn et al., 2012**	USA	1,019	No	Probit regression	Yes
**Fan et al., 2014**	USA	403,305	No	Multilevel regression models	Yes
**Frankenfeld et al., 2015**	USA	USA population	No	Linear regression	Yes
**Fuller et al., 2013**	USA	1,440	Yes	Bivariate linear regression	No
**Gartin 2012**	Paraguay	126	No	Linear regression no GIS analysis	No
**Ghosh-Dastidar et al., 2014**	USA	1,214	No	Multivariate logistic regression	Yes
**Hanibuchi et al., 2011**	Japan	39,765	No	Multiple linear regression logistic regression	Yes
**Hattori et al., 2013**	USA	97,678	Yes	Negative binomial regression OLS and logistic regression	Yes
**Hobbs 2017**	UK	4,723	No	Single-level linear regression linear multilevel	No
**Hobbs et al., 2019**	UK	22,889	No	Binary logistic regression	No
**Hobbs et al., 2019**	UK	7,544	No	Structural equation modelling	No
**Hollands et al., 2013**	Canada	1,269 geographic areas	Yes	OLS and spatial auto-regressive error	Yes

Reference	Country	Sample population	Physical activity consideration	Statistical Method	Significant findings
**Hollands et al., 2014**	Canada	84,341	Yes	Multivariable regression analyses	Yes
**Hosler et al., 2009**	USA	Columbia and Greene counties in NY	No	Bivariate correlations	No
**Inagami et al., 2009**	USA	2,156	No	Multilevel modelling	Yes
**Jeffery et al., 2006**	USA	1,033	Yes	Logistic regression	No
**Jilcott et al., 2011**	USA	USA population	Yes	Multilevel linear models	Yes
**Kruger et al., 2014**	USA	1,345	No	Stepwise linear regressions	Yes
**Laxy et al., 2015**	USA	1,570	Yes	Multivariate linear and logistic regression	No
**Li et al., 2008**	USA	1,221	Yes	Linear and logistic regression	No
**Li et al., 2009b**	USA	1,145	Yes	Multilevel Poisson regression	Yes
**Li et al., 2009c**	USA	1,145	Yes	Multilevel	Yes
**Liese 2017**	USA	459	Yes	Multivariable/hierarchical linear regression	Yes
**Lopez 2007**	USA	15,358	No	Multilevel logistic regression	Yes
**Macdonald et al., 2011**	Scotland	991	No	Multilevel	Yes
**Mackenbach 2019**	Belgium France Hungary Netherlands and the United Kingdom.	5,076	No	Logistic and linear regressions	No
**Mackenbach 2019**	Netherlands	2,812	No	Linear and multinomial logistic regression	Yes
**Maddock 2004**	USA	USA population	Yes	Logistic regression and multinomial regression	Yes
**Mason 2018**	UK	40,1435	Yes	Multilevel linear regression	Yes

Reference	Country	Sample population	Physical activity consideration	Statistical Method	Significant findings
**Mazidi and Speakman 2017**	USA	2,996 counties. 3,138 counties USA population~ 170 million adults	Yes	Linear regression	No
**Mehta and Chang 2008**	USA	876,091	Yes	Bivariate correlation hierarchical multiple regression	Yes
**Mejia et al., 2015**	USA	5,185	Yes	Multilevel analysis Two-level hierarchic regression models	Yes
**Mendes et al., 2013**	Brazil	3,404	No	Negative binomial	Yes
**Michimi and Wimberly 2010**	USA	1,477,828	No	Poisson regression model in a single level	No
**Michimi and Wimberly 2015**	USA	300,933	No	Latent class analysis	Yes
**Morland et al., 2006**	USA	10,763	Yes	Multilevel logistic regression	Yes
**Murphy 2017**	Australia	3,218	No	Generalised estimating equations	Yes
**Murphy 2018**	Australia	3,141	Yes	Generalized estimating equations models	Yes
**Mylona 2020**	USA	20,927	Yes	Multivariate logistic regression	Yes
**Oexle et al., 2015**	USA	838	No	Multilevel logistic regression	Yes
**Oka et al., 2013**	USA	5,485	Yes	Multilevel modelling binomial regression	Yes
**Patel et al., 2017**	India	1,782	Yes	Logistic regression	Yes
**Pearce et al., 2009**	New Zealand	12,529	No	Multinomial Logistic regression	No
**Pineda et al., 2021**	Mexico	37,969	Yes	Multilevel linear regression	Yes
**Polsky et al., 2016**	Canada	10,199	Yes	logistic regression stratified linear regression	Yes
**Polsky et al., 2016**	Canada	10,199	Yes	Multilevel modelling	No

Reference	Country	Sample population	Physical activity consideration	Statistical Method	Significant findings
**Pouliou and Elliott 2010**	Canada	115,548	Yes	Two-level logistic regression multilevel	Yes
**Prince et al., 2012**	Canada	4,727	Yes	Logistic and linear regression	Yes
**Pruchno et al., 2014**	USA	5,688	No	Multivariate analysis	No
**Richardson et al., 2015**	USA	5,114	Yes	Binomial multivariate multilevel model	Yes
**Rundle et al., 2009**	USA	13,102	Yes	Multilevel structural equation	Yes
**Salois 2012**	USA	3,051 counties	Yes	Structural equation model	Yes
**Singleton et al., 2016**	USA	3,135	No	Linear regression	Yes
**Slack et al., 2014**	USA	3,109 counties	Yes	Multilevel analysis	Yes
**Spence et al., 2009**	Canada	2,900	No	Robust regression MM estimator regression	Yes
**Stark et al., 2013**	USA	48,482	Yes	OLS spatial regression	No
**Tung et al., 2016**	USA	267	Yes	Linear regression	No
**Viola et al., 2013**	USA	48,014	Yes	Weighted logistic regression models	Yes
**Walker 2020**	Canada	8,076	Yes	Logistic regression	Yes
**Wang et al., 2007**	USA	7,595	Yes	Multilevel Pearson correlation coefficients	Yes
**Xu and Wang 2015**	USA	328,156	Yes	Bayesian ecologic approach for spatial prediction	Yes
**Yan et al., 2015**	USA	3,041	No	Multilevel	No
**Yenerall 2017**	USA	784	No	Logistic regression model	No
**Zhang 2019**	USA	8,365	No	Generalised linear regression and logistic regression	No
**Zhang 2020**	China	170,872	Yes	Five-level logistic regression models	Yes
**Zick et al., 2009**	USA	898,387	Yes	Multilevel Logistic model	Yes
**Jaime et al., 2011**	Brazil	2,122	Yes	Correlation analyses Pearson	No

Reference	Country	Sample population	Physical activity consideration	Statistical Method	Significant findings
				correlation coefficient	
**Longitudinal studies**					
**Block et al., 2011a**	USA	3,113	No	Cross-classified multilevel model	No
**Boone-Heinonen et al., 2013**	USA	4,092	Yes	Regression with fixed effects for individuals	Yes
**Burgoine et al., 2011**	England	893	Yes	Correlation ANOVA and Chi-square analysis	No
**Burgoine et al., 2016**	UK	2,039	Yes	Linear and logistic regression	Yes
**Carroll 2020**	Australia	2,253	Yes	Spearman rank correlation latent variable growth models	Yes
**Du et al., 2014**	China	24,396	Yes	Random intercept-slope growth model	Yes
**Dubowitz et al., 2012**	USA	60,775	Yes	Logistic regression multivariate regression models with random effects	Yes
**Gibson et al., 2011**	USA	27,825	Yes	OLS	Yes
**Hobbs 2019**	UK	8,864	No	Multilevel linear regression	No
**Jilcott Pitts et al., 2015**	USA	205	No	Multiple linear and logistic regression	No
**Meyer et al., 2015**	USA	14,397	Yes	OLS logistic regression	No
**Rummo 2017**	USA	12,174	Yes	Instrumental-variables regression	No
**Xu et al., 2013**	China	28,063	Yes	Bayesian hierarchical regressions multilevel	Yes
**Zenk 2017**	USA	219	No	Hierarchical linear regression	No

GLM: General linear model, OLS: ordinary least squares regression, ANOVA: analysis of variance

Table S4. Food environment characteristics and methods of the reviewed studies

Reference	Geographic unit of observation	Store type	Spatial food store measure	Buffer size	Food outlet source
**Abbott et al., 2014**	Suburbs	Green grocers, supermarkets, FF	Density	NA	NA
**Adachi-Mejia 2017**	Neighbourhood	FF, restaurant, GS, supermarket	Density and proximity	1 km street-network	Government and commercial
**Ahern et al., 2011**	County	Direct-to-consumer farms, GS, restaurants, FF, CS	Density	NA	Government
**Albalawi 2020**	Postcode district (PD)	FF, restaurants, delivery, takeaways, pubs, and cafes	Density	Postcode district	Government and Commercial databases
**Backes 2019**	residential address	Supermarkets, GC, CS	Density	400m radius buffer around residential locations	Systematic survey
**Block et al., 2011a**	CTA	FF, restaurants, bakeries/coffee shops, supermarkets, GS, CS	Proximity (driving time)	NA	Government, Yellow pages, Commercial
**Bodor et al., 2010**	CTA	Supermarkets, medium food stores, small food stores, CS, general merchandise stores, FF	Density	2km	Government
**Boone-Heinonen et al., 2013**	Census block group	FF, supermarkets, CS	Proximity	1km Euclidean	Commercial
**Burgoine 2017**	Residential address	Supermarkets	Proximity	Street network proximity	Government
**Burgoine 2018**	Residential address	FF	%	1-mile straight-line radius (circular) residential address	Government
**Burgoine et al., 2011**	LSOA	FF, supermarkets, greengrocer, CS, pizza delivery, takeaway	Density	NA	Yellow pages
**Burgoine et al., 2016**	residential and work address	FF, supermarkets	Density	1-mile straight-line radius (circular) buffers, cantered on home and work addresses	Government
**Carroll 2020**	Census collection districts	FF; RFEI	Density, RFEI	1600m road-network proximity buffers from participants' residence	Government and commercial databases

Reference	Geographic unit of observation	Store type	Spatial food store measure	Buffer size	Food outlet source
**Cerin et al., 2011**	CTA	FF, CS, restaurants, gas/non gas food marts	Density, proximity, diversity	1km residential, 1km network	Government, Yellow Pages, online business, directories, and ground-truthing
**Chaparro et al., 2017**	CTA	FF, CS, LS	Density	NA	Government
**Chen et al., 2010**	County	Chain grocers	Density	1-mile	Government
**Chen et al., 2013**	CTA	Restaurants, FF	Density, proximity	0·5 mile	Government
**Chen et al., 2016**	County, food desert tract	Supermarkets, GS, clubs and supercentres, CS, specialty food stores, pharmacies, restaurants	Density	NA	Government
**Chen 2019**	CTA	mRFEI	mRFEI	None	Government
**Chen 2020**	CTA	mRFEI	mRFEI	None	Government
**Christian, 2012**	CTA	Supermarkets, CS, FV markets, limited-service restaurants	Density	0·50 miles Euclidian	Government
**Conroy 2018**	Census block group	REI, RFEI	REI, RFEI	1·6 km pedestrian network	Government and commercial databases
**Crawford et al., 2008**	State	FF chains	Proximity, density	2km	Yellow pages, commercial/online
**Crawford et al., 2008**	Residential address	FF	Density, proximity	2km	Government
**Dake et al., 2016**	Enumeration areas	Out-of-home cooked food, CS, FV	Density	NA	Ground truthing
**Drewnowski et al., 2012**	County and block group code	Supermarkets	Network proximity (3 types, driving)	NA	Field work
**Drewnowski et al., 2014a**	County	Supermarkets	Network proximity	Buffers cantered on each study participant's residence·	Government
**Du et al., 2014**	Neighbourhood	FF, indoor restaurants, and	Density	NA	Community questionnaire

Reference	Geographic unit of observation	Store type	Spatial food store measure	Buffer size	Food outlet source
		fixed outdoor food stalls			, community leader
**Dubowitz et al., 2012**	Tract and metropolitan statistical area	GS, supermarkets, FF	Density	0·75, 1·5, and 3·0 miles	Yellow pages, commercial
**Dunn et al., 2012**	CTA	FF	Density	1, 3 miles	Ground-truthing
**Fan et al., 2014**	Census block group, CTA, zip code, 1 km buffer around residential address.	GS, CS, restaurants	Density	1km	Commercial
**Frankenfeld et al., 2015**	Block group	FF, CS and pharmacies, GS, and specialty food stores	Ratio unhealthy: healthy food outlets, k-mean cluster	NA	Commercial
**Fuller et al., 2013**	CTA	Primary food stores	Road network proximity	NA	Commercial and participant survey
**Gartin, 2012**	Census block group	Open-air markets, CS, supermarkets	Proximity	0·5-1 miles	Ground-truthing
**Ghosh-Dastidar et al., 2014**	CTA	Supermarkets, specialty grocery stores, discount grocery stores, supercentres, meat/seafood markets, wholesale clubs	Network proximity	NA	Geocoded from participant's interview
**Gibson, 2011**	Zip code	GS, CS, restaurants	Density	NA	Government
**Hanibuchi et al., 2011**	Block	CS, FF, supermarkets	Radial proximity based on street network	500m	Yellow pages
**Hattori et al., 2013**	Residential address	FF, restaurants, CS, small food stores, GS, supermarkets	Density, Euclidian proximity within walking proximity	0·25, 0·5, 1-, 1·5-, and 3-miles circular buffers	Commercial
**Hobbs 2017**	Residential address	Supermarkets, takeaways, CS	Density	800m and 2000m) residential address; LSOA (km^2)	Government and commercial databases

Reference	Geographic unit of observation	Store type	Spatial food store measure	Buffer size	Food outlet source
**Hobbs 2019**	Residential postcode	FF, LS, CS, and restaurants	Density	2km	Commercial database
**Hobbs 2019**	Residential postcode	FF	Density	2km radial buffers around home postcode	Commercial database
**Hobbs 2019**	Cluster of food outlets	FF, LS, restaurants, CS, independent supermarkets, specialty, café	Density	1600m Euclidean radial buffer	National database
**Hollands et al., 2013**	Forward sortation area	FF, coffee outlets, restaurants	Density	NA	Commercial
**Hollands et al., 2014**	Forward sortation area	FF, restaurants	Density	NA	Commercial
**Hosler, 2009**	Census block group	Food stores, farmer's markets	Density, Euclidian proximity	NA	Research centre
**Inagami et al., 2009**	CTA	FF	Density	NA	Government
**Jaime et al., 2011**	Sub-municipality	Supermarket, GS, FV, FF chains	Density	NA	Government and commercial
**Jeffery et al., 2006**	Not defined	FF, restaurants	Proximity, density, frequency	Radio of 0·5 miles, 1 mile and 2 miles (home and work addresses)	Government and commercial
**Jilcott et al., 2011**	County	Farmers' markets, GS, supermarkets, supercentres	Density	NA	Government
**Jilcott Pitts et al., 2015**	County	Farmers' market	Network proximity	NA	Ground truthing
**Kruger et al., 2014**	County	FF	Proximity	NA	Yellow pages and internet
**Laxy et al., 2015**	Neighbourhood	FF, CS, supermarkets	Network proximity	NA	Commercial
**Li et al., 2008**	Census block group	FF	Density	NA	Commercial
**Li et al., 2009b**	Census block group	FF	Density	NA	Government and commercial
**Li et al., 2009c**	Census block group	FF	Density	NA	Government and commercial
**Liese 2017**	Residential address	Supermarkets, supercentre, or warehouse club	Proximity	None	Ground-truthing survey and government

Reference	Geographic unit of observation	Store type	Spatial food store measure	Buffer size	Food outlet source
**Lopez, 2007**	Zip code	Supermarkets, FF, general retail	Density	NA	Government and commercial
**Macdonald et al., 2011**	Postal code	Supermarkets, independent food stores and chain outlets, FV	Network Proximity	500m and 1000m	Government
**Mackenbach 2019**	Neighbourhood	FF	Spatial access	300m Euclidean buffer around selected residential neighbourhoods	Commercial database
**Mackenbach 2019**	Residential address	FF	Spatial access	300m Euclidean buffers around participants' home addresses	Commercial database
**Maddock, 2004**	State	FF	Square miles per food outlet	NA	Yellow pages and online verification
**Mason 2018**	Residential address	FF	Street-network Proximity	500m, 500–999m, 1000–1999m, 2000m from residential address	National database
**Mazidi and Speakman, 2017**	County	FF and restaurants	Density	NA	Government
**Mehta and Chang, 2008**	County	FF	Density	NA	Government
**Mejia et al., 2015**	Neighbourhood	FF, CS, small food stores, GS, supermarkets.	Density, Euclidian proximity	0·25, 0·5, 1, 1·5, and 3 miles	Commercial
**Mendes et al., 2013**	Postal code	Supermarkets, hypermarkets	Presence, health vulnerability index	NA	Commercial
**Meyer et al., 2015**	Neighbourhood	CS, coops/natural food stores, specialty markets, supermarkets, GS, FF, food stances/cafeterias, restaurants	Density	3km	Commercial
**Michimi and Wimberly, 2010**	County	Supermarkets and supercentres other grocery stores (except convenience stores), and warehouse clubs	Euclidian proximity	NA	Government

Reference	Geographic unit of observation	Store type	Spatial food store measure	Buffer size	Food outlet source
**Michimi and Wimberly, 2015**	County	Supermarkets, snack/coffee shops, restaurants, FF, CS	Factor analysis	NA	Government
**Morland et al., 2006**	CTA	Supermarkets, grocery stores, or smaller non–corporate-owned food stores, CS, restaurants, FF, and limited-service restaurant	Density	NA	Government
**Murphy 2017**	Residential address	Supermarkets	Density, proximity	800, 1000, 1600, 2000 and 3000m network	Government, commercial, and ground truthing
**Murphy 2018**	Residential address	FF, supermarkets	Density, proximity	800m, 1000m, 1600m, 2000m, and 3000m network	Government and commercial
**Mylona 2020**	Residential address	FF	kernel Density	3·1 km	Commercial database
**Oexle et al., 2015**	County	FF	Count, proximity	1 mile	Ground truthing
**Oka et al., 2013**	CTA	Food markets, CS, grocers, restaurants, pizza stores, gyms	Principal component analysis	NA	Government
**Patel et al., 2017**	census enumeration blocks	FF, restaurants	Density	1-km	Ground truthing
**Pearce et al., 2009**	Census mesh block	Multinational/local FF	Travel proximity	NA	Yellow pages
**Pineda et al.., 2021**	CTA	FF, restaurant, CS, supermarkets	Density	NA	Government and ground truthing
**Polsky et al., 2016**	Census block group	FF, restaurants	Absolute density, relative density	720m along the street network	Commercial
**Polsky et al., 2016**	Dissemination blocks	Restaurants	Absolute and relative Density	720m	Commercial
**Pouliou and Elliott, 2010**	Postal code	FF, CS, GS, supermarkets	Density	1km	Government
**Prince et al., 2012**	Neighbourhood	GS, CS, specialty food stores, FF, restaurants	Density	NA	Another study

Reference	Geographic unit of observation	Store type	Spatial food store measure	Buffer size	Food outlet source
**Pruchno et al., 2014**	CTA	FF, store fronts, supermarkets, GS, CS	Density	NA	Government
**Richardson et al., 2015**	Residential address	FF, restaurants, supermarkets, CS	Density	3 and 8km Euclidean	Commercial
**Rummo 2017**	Residential address	FF, restaurants, CS, GS, supermarkets	percentage of CS, GS, and S out of sum of them, percentage of FF and FSR out of the sum of them	1-km street network proximity from respondents' residences	Commercial database
**Rundle et al., 2009**	Network buffer around residential address	Supermarkets, FV markets, FF, pizzerias, bakeries, and candy and nut stores	Density	805m network	Commercial
**Salois, 2012**	County	Restaurants, FF, supermarkets, GS, gas-based CS, no-gas CS, super centre/warehouse club stores, farmers' markets	Density	NA	Government
**Singleton et al., 2016**	County	GS, CS, farmer's markets, supercentres, supermarkets, FF, restaurants	Density	NA	Government
**Slack et al., 2014**	County/food desert tract	FF	Density	NA	Government
**Spence et al., 2009**	Postal code	Supermarkets, FF, specialty food stores, CS	Count, ratio, Retail Food Environment Index (RFEI)	800m and 1600m	Government and commercial
**Stark et al., 2013**	Zip code	GS, supermarkets, national/local FF, pizza restaurants, CS, Food outlets were categorized, bodegas, bakeries, candy and nut stores, meat markets, restaurants, GS, fish markets and specialty food stores	Density, relative concentration (proportion), diversity	NA	Commercial
**Tung et al., 2016**	Neighbourhood	GS	Euclidian Proximity	NA	Self-reported

Reference	Geographic unit of observation	Store type	Spatial food store measure	Buffer size	Food outlet source
**Viola et al., 2013**	Postal code	FF, pizzerias, supermarkets	Density, Network Proximity	¼ miles	Commercial
**Walker 2020**	Residential postcode	FF, restaurants, pubs to liquor stores, markets	ratio (FF/FSR, pubs/liquor stores); Count (markets)	500m	Ground-truthing survey
**Wang et al., 2007**	Census tracts and/or block groups	FF, food retail stores	Density, Euclidian Proximity	0·5 miles	Government and commercial
**Xu and Wang, 2015**	County	FF and restaurants	Ratio FF:Restaurants	NA	Government
**Xu et al., 2013**	Street committee	FF, restaurants	Density	720m street network	Government
**Yan et al., 2015**	County	Supercentres, GS, CS, specialized food stores	Density	NA	Government
**Yenerall 2017**	Residential address	GS	accessibility (network Proximity)	None	National database
**Zenk 2017**	Census block group	CS, small GS, large GS, liquor stores	availability (Count)	0·5-mile radial buffer around participants' census block centroid	Ground-truthing survey
**Zhang 2019**	CTA	mRFEI	mRFEI	0·5 miles	Commercial database
**Zhang 2020**	Subdistrict/town	FF, restaurants, small GS, LS	Density	NA	National database
**Zick et al., 2009**	Census block group	Healthy grocery stores, CS, restaurants, FF, multiple retail food options	Density, diversity, and design	NA	Government and commercial

CS: convenience store, FF: fast-food outlet, FV: fruit and vegetable store, GS: grocery store, LS: liquor store, RFEI: retail food environment index, mRFEI: modified retail food environment index. NA: not available/not applicable

5. Risk of bias assessment

Table S5. Risk of bias and quality assessment of reviewed studies

Reference	Population sampling	Outcome	Exposure (food environment)	Food outlet data source	Spatial analysis method	PA	Study design	Statistical Method	Data temporality/ time match	Score
**Abbott et al., 2014**	Low	High	Low	High	High	High	High	High	High	2
**Adachi-Mejia et al., 2017**	High	High	Low	Low	Low	Low	High	High	High	4
**Ahern et al., 2011**	Low	High	Low	High	High	Low	High	High	High	3
**Albalawi 2020**	High	Low	Low	Low	High	High	High	High	High	3
**Backes 2019**	Low	High	Low	Low	High	High	High	High	Low	4
**Block et al., 2011a**	Low	Low	Low	High	High	High	Low	Low	Low	6
**Bodor et al., 2010**	High	High	Low	Low	High	High	High	High	Low	3
**Boone-Heinonen et al., 2013**	Low	Low	Low	High	High	Low	Low	High	Low	6
**Burgoine 2017**	Low	Low	High	Low	High	Low	High	High	High	3
**Burgoine 2018**	Low	Low	Low	Low	High	Low	High	High	High	4
**Burgoine et al., 2011**	Low	High	Low	High	High	Low	Low	High	Low	5
**Burgoine et al., 2016**	Low	Low	High	Low	Low	High	High	High	Low	5
**Carroll 2020**	Low	Low	Low	Low	Low	Low	Low	High	Low	7
**Cerin et al., 2011**	Low	High	Low	Low	Low	Low	High	Low	High	6
**Chaparro et al., 2017**	Low	High	High	High	Low	High	High	Low	Low	4
**Chen et al., 2010**	Low	High	High	Low	High	High	High	Low	High	3
**Chen et al., 2013**	High	High	High	Low	Low	Low	High	Low	Low	5
**Chen et al., 2016**	Low	High	High	High	High	High	High	Low	High	2
**Chen et al., 2019**	High	Low	Low	Low	High	High	High	Low	High	4
**Chen et al., 2020**	High	Low	High	Low	High	High	High	High	High	2

Reference	Population sampling	Outcome	Exposure (food environment)	Food outlet data source	Spatial analysis method	PA	Study design	Statistical Method	Data temporality/ time match	Score
**Christian, 2012**	High	High	Low	Low	High	High	High	High	Low	3
**Conroy 2018**	High	High	Low	Low	High	High	High	High	High	2
**Cooksey-Stowers et al., 2017**	Low	High	Low	High	Low	Low	High	High	Low	5
**Crawford et al., 2008**	High	High	High	High	High	Low	High	High	High	1
**Dake et al., 2016**	High	Low	Low	High	Low	Low	High	Low	Low	6
**Drewnowski et al., 2014b**	High	High	High	Low	High	High	High	Low	Low	3
**Drewnowski, 2012**	High	High	High	Low	High	High	High	High	Low	2
**Du et al., 2014**	Low	Low	Low	High	Low	Low	Low	High	High	6
**Dubowitz et al., 2012**	Low	Low	Low	Low	High	Low	Low	High	High	6
**Dunn et al., 2012**	Low	High	High	Low	High	High	High	Low	Low	4
**Fan et al., 2014**	Low	High	Low	High	High	High	High	High	High	2
**Frankenfeld et al., 2015**	Low	High	Low	High	High	High	High	High	High	2
**Fuller et al., 2013**	High	High	High	Low	High	Low	High	Low	Low	4
**Gartin, 2012**	High	Low	Low	Low	High	High	High	High	Low	4
**Ghosh-Dastidar et al., 2014**	Low	Low	Low	Low	Low	High	High	High	Low	6
**Gibson, 2011**	Low	Low	Low	Low	High	High	High	High	Low	5
**Hanibuchi et al., 2011**	Low	High	Low	High	High	High	High	High	High	2
**Hattori et al., 2013**	Low	High	Low	High	Low	Low	High	Low	High	5
**Hobbs 2017**	High	High	Low	Low	Low	High	High	High	Unclear	3
**Hobbs 2019**	High	High	Low	Low	High	High	Low	Low	High	4
**Hobbs 2019**	High	High	High	Low	High	High	High	High	High	1
**Hobbs 2019**	High	High	High	Low	High	High	High	High	Unclear	1

Reference	Population sampling	Outcome	Exposure (food environment)	Food outlet data source	Spatial analysis method	PA	Study design	Statistical Method	Data temporality/ time match	Score
**Hollands et al., 2013**	High	High	Low	Low	High	Low	High	Low	Low	5
**Hollands et al., 2014**	Low	High	High	Low	High	Low	High	Low	Low	5
**Hosler, 2009**	Low	High	High	Low	High	High	High	High	High	2
**Inagami et al., 2009**	Low	High	High	Low	High	High	High	Low	High	3
**Jaime et al., 2011**	Low	High	Low	Low	High	Low	High	High	Low	5
**Jeffery et al., 2006**	Low	High	High	High	Low	Low	High	High	High	3
**Jilcott et al., 2011**	Low	High	Low	Low	High	Low	High	Low	High	5
**Jilcott Pitts et al., 2015**	High	High	High	Low	Low	High	High	High	Low	3
**Kruger et al., 2014**	High	High	High	High	High	High	High	High	High	0
**Laxy et al., 2015**	Low	Low	Low	High	Low	Low	High	High	Low	6
**Li et al., 2008**	Low	High	High	High	High	Low	High	Low	Low	4
**Li et al., 2009c**	Low	High	High	High	High	Low	High	Low	Low	4
**Li et al., 2009c**	Low	High	High	High	High	Low	High	Low	Low	4
**Liese 2017**	High	Low	Low	Low	High	Low	High	High	Low	4
**Lopez, 2007**	Low	High	Low	High	High	High	High	Low	High	3
**Macdonald et al., 2011**	Low	High	Low	Low	High	High	High	High	High	3
**Mackenbach 2019**	High	High	High	Low	Low	High	High	Low	High	3
**Mackenbach 2019**	High	High	High	Low	High	High	High	High	Low	2
**Maddock, 2004**	Low	High	High	High	High	Low	High	Low	High	3
**Mason 2018**	High	Low	High	Low	High	Low	High	High	Unclear	2
**Mazidi and Speakman, 2017**	Low	High	High	Low	Low	Low	High	High	Low	5
**Mehta and Chang, 2008**	Low	High	High	High	High	High	High	High	Low	2

Reference	Population sampling	Outcome	Exposure (food environment)	Food outlet data source	Spatial analysis method	PA	Study design	Statistical Method	Data temporality/ time match	Score
**Mejia et al., 2015**	High	High	Low	Low	Low	Low	High	High	High	4
**Mendes et al., 2013**	High	High	High	High	High	High	High	High	High	0
**Meyer et al., 2015**	High	Low	Low	High	High	Low	Low	High	High	4
**Michimi and Wimberly, 2010**	High	High	Low	High	High	High	High	High	Low	2
**Michimi and Wimberly, 2015**	High	High	Low	Low	High	High	High	Low	Low	4
**Morland et al., 2006**	Low	Low	Low	High	High	Low	High	Low	High	5
**Murphy 2017**	High	High	High	Low	High	High	High	Low	Low	3
**Murphy 2018**	High	High	High	Low	High	Low	High	Low	Unclear	2
**Mylona 2020**	High	Low	High	Low	High	Low	High	Low	Unclear	3
**Oexle et al., 2015**	High	High	High	Low	Low	High	High	High	High	2
**Oka et al., 2013**	Low	Low	Low	High	High	Low	High	Low	High	5
**Patel et al., 2017**	Low	Low	High	Low	Low	Low	High	High	High	5
**Pearce et al., 2009**	Low	Low	High	High	High	High	High	Low	High	3
**Pineda et al., 2021**	Low	Low	Low	Low	Low	Low	High	Low	High	7
**Polsky 2016**	High	High	Low	Low	Low	Low	High	High	High	3
**Polsky et al., 2016**	Low	Low	High	High	High	Low	High	High	High	3
**Pouliou and Elliott, 2010**	Low	High	Low	High	High	Low	High	High	High	3
**Prince et al., 2012**	Low	High	Low	High	High	Low	High	Low	High	4
**Pruchno et al., 2014**	High	High	Low	High	High	High	High	Low	Low	3
**Richardson et al., 2015**	High	Low	High	High	High	Low	Low	Low	Low	5
**Rummo 2017**	High	Low	Low	Low	High	Low	Low	High	Low	5
**Rundle et al., 2009**	Low	Low	Low	High	High	Low	High	Low	High	5

Reference	Population sampling	Outcome	Exposure (food environment)	Food outlet data source	Spatial analysis method	PA	Study design	Statistical Method	Data temporality/ time match	Score
**Salois, 2012**	Low	High	Low	High	High	Low	High	High	Low	4
**Singleton et al., 2016**	High	High	Low	Low	Low	High	High	High	High	
**Slack et al., 2014**	Low	High	High	High	High	Low	High	Low	High	3
**Spence et al., 2009**	High	High	Low	High	Low	High	High	High	High	2
**Stark et al., 2013**	High	High	Low	High	Low	Low	High	Low	Low	5
**Tung et al., 2016**	High	Low	High	High	High	Low	High	High	Low	3
**Viola et al., 2013**	High	High	Low	High	Low	Low	High	Low	Low	5
**Walker 2020**	High	Low	Low	Low	Low	Low	High	Low	Unclear	5
**Wang et al., 2007**	Low	High	Low	High	Low	Low	High	Low	High	5
**Xu and Wang, 2015**	Low	High	High	High	High	Low	High	Low	Low	4
**Xu et al., 2013**	High	High	High	Low	Low	Low	Low	Low	Low	6
**Yan et al., 2015**	High	High	Low	High	High	High	High	High	High	1
**Yenerall 2017**	Low	High	High	Low	High	High	High	High	Unclear	2
**Zenk 2017**	High	Low	Low	Low	High	High	Low	High	Low	5
**Zhang 2019**	High	Low	Low	Low	High	High	High	High	High	3
**Zhang 2020**	High	Low	Low	Low	High	Low	High	High	Low	4
**Zick et al., 2009**	High	High	Low	High	Low	Low	High	Low	High	4

A high score represents a lower risk of bias while a low score indicates a higher risk of bias. The maximum possible score was 9.

6. Food outlet density and proximity and its association with BMI

Figure S2. Fast-food outlet density and proximity and its association with BMI

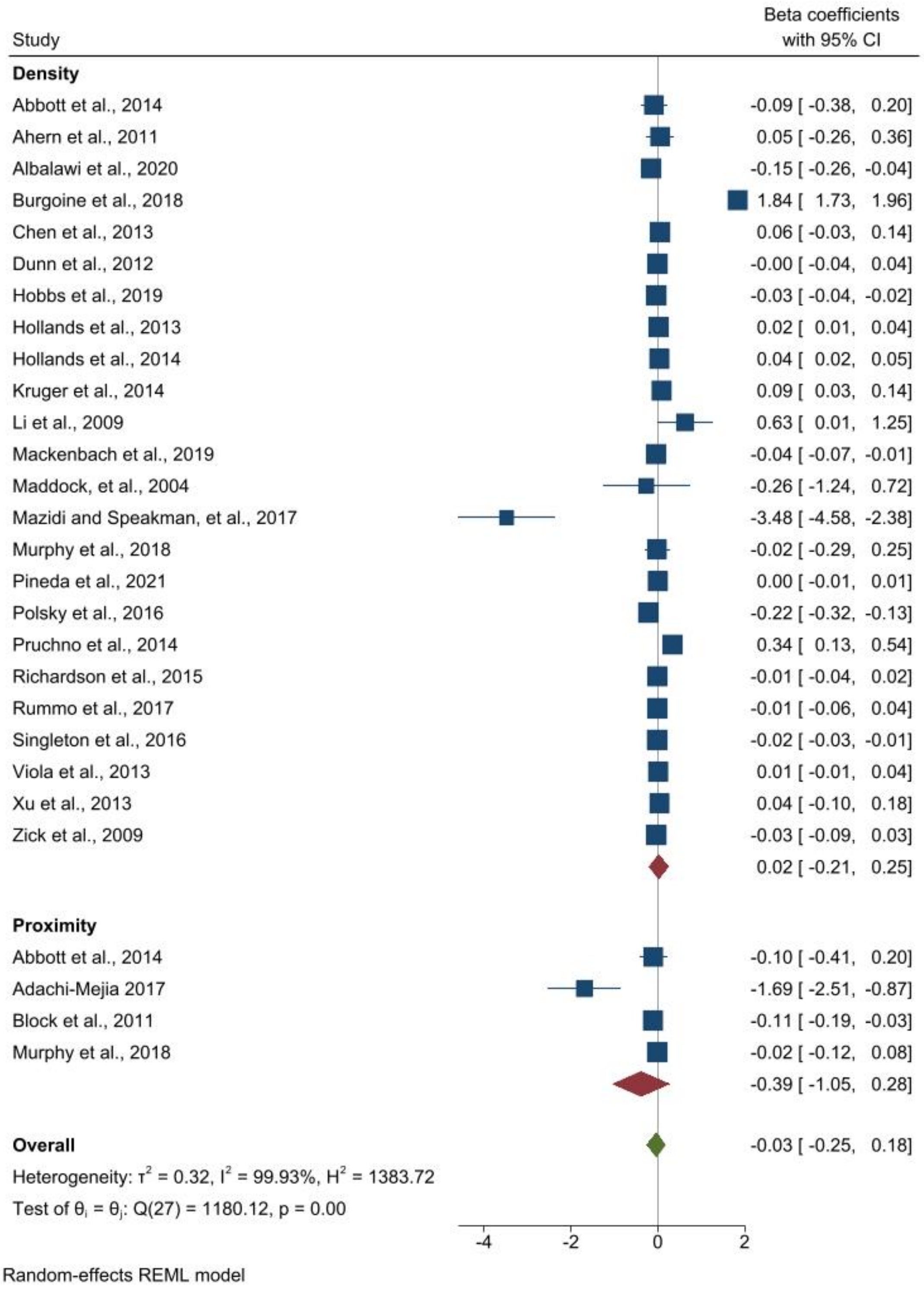

Figure S3. Restaurant density and its association with BMI.

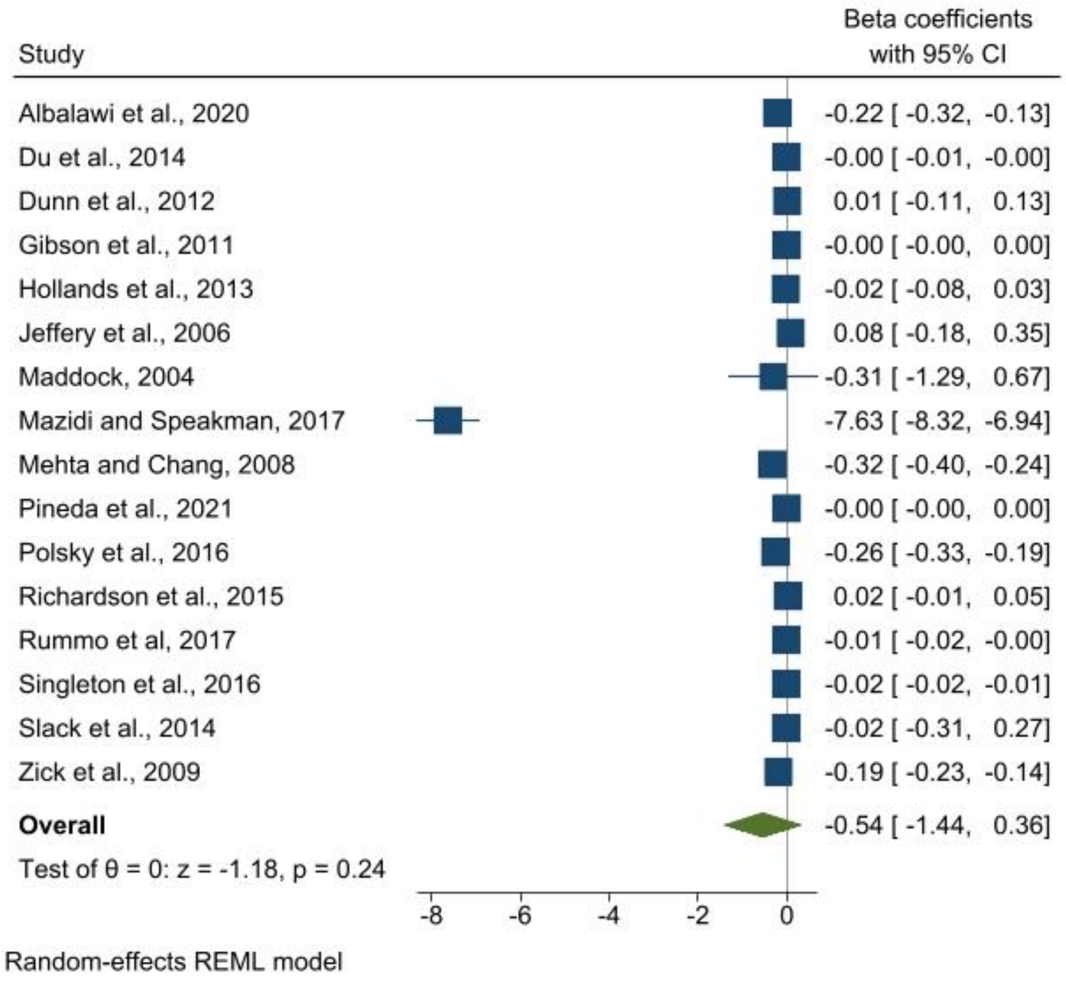

Figure S4. Convenience store density and proximity and its association with BMI.

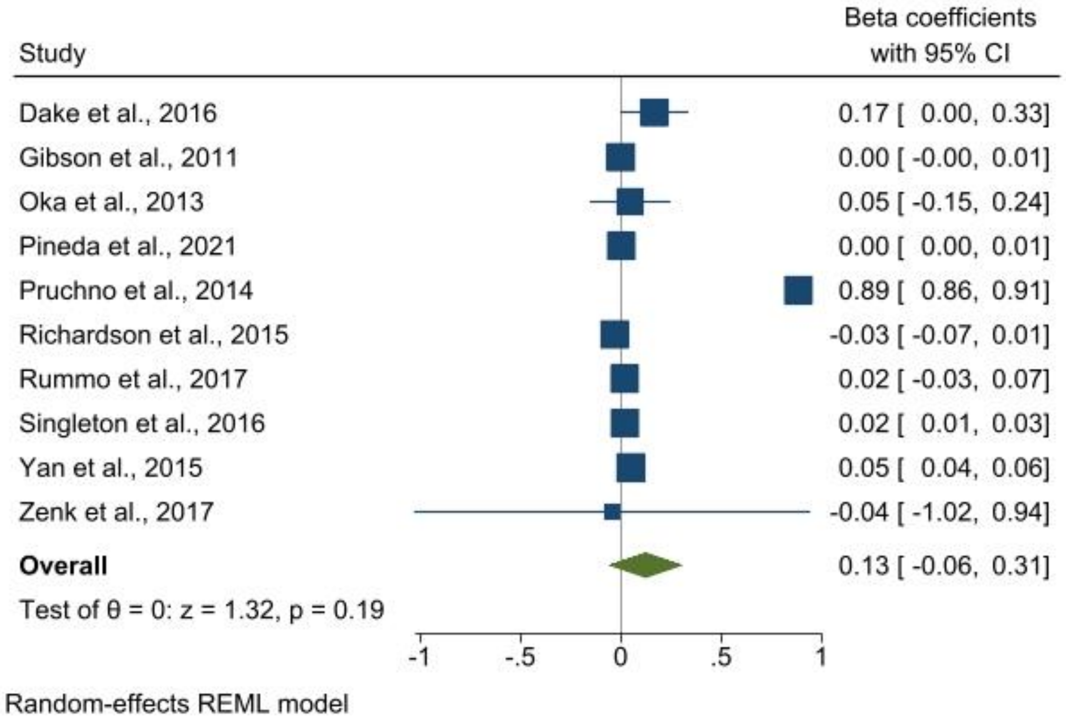

Figure S5. Supermarket density and proximity and its association with BMI.

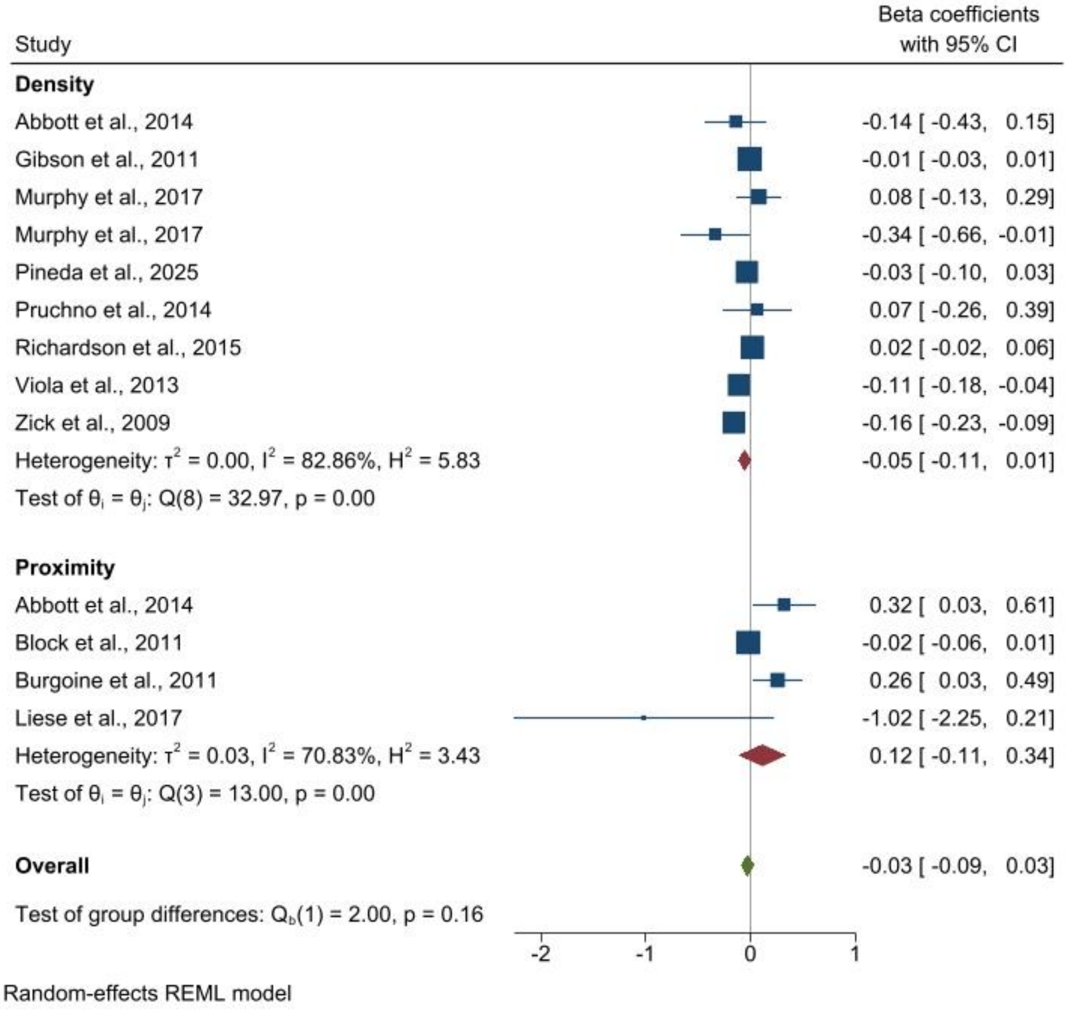

Figure S6. Fruit and vegetable store density and its association with BMI.

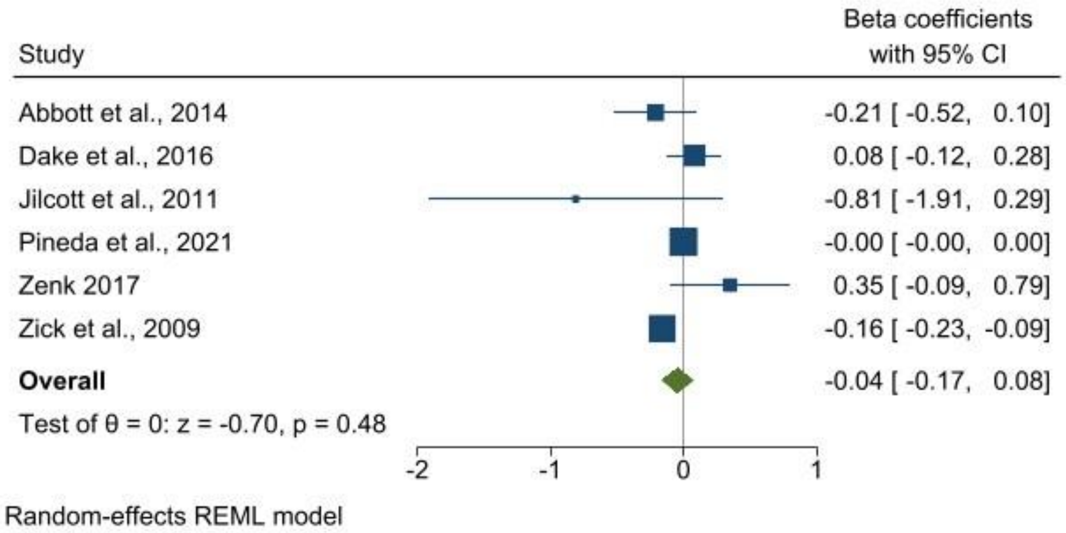

Figure S7. Retail food environment index (RFEI) and its association with BMI.

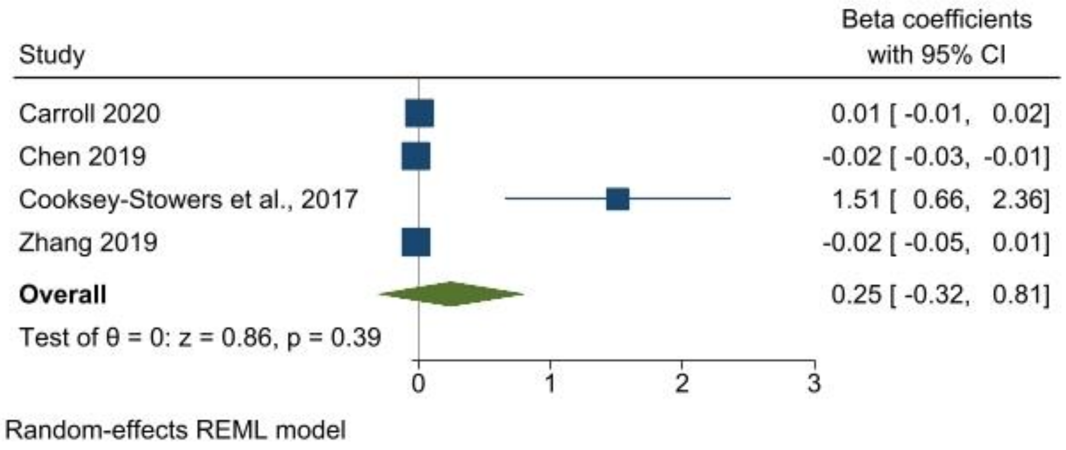

Supplement: Supplementary data [file bmjnph-2023-000663supp001.pdf]
